# Impact of omicron wave and associated control measures in Shanghai on health management and psychosocial well-being of patients with chronic conditions

**DOI:** 10.1515/med-2023-0674

**Published:** 2023-03-28

**Authors:** Zhimin Xu, Gabriela Lima de Melo Ghisi, Xia Liu, Lixian Cui, Sherry L. Grace

**Affiliations:** Xinhua Hospital Affiliated to Shanghai Jiaotong University School of Medicine, Shanghai, 200082, China; Cardiovascular Prevention and Rehabilitation Program, Toronto Rehabilitation Institute, University Health Network, Toronto, M4G 2R6, Canada; Chengdu Wanda UPMC Hospital, Chengdu, 610218, Sichuan Province, China; Division of Arts and Sciences, NYU Shanghai, 200122, Shanghai, China; Faculty of Health, York University, 4700 Keele St, North York, M3J 1P3, Canada; KITE-Toronto Rehabilitation Institute & Peter Munk Cardiac Centre, University Health Network, University of Toronto, Toronto, Canada

**Keywords:** COVID-19, psychological distress, adaptation, psychological, mental health, self-management, chronic disease, surveys and questionnaires

## Abstract

The objective of this cross-sectional study was to investigate health management, well-being, and pandemic-related perspectives of chronic disease patients in the context of stringent measures, and associated correlates. A self-report survey was administered during the Omicron wave lockdown in Shanghai, China. Items from the Somatic Symptom Scale (SSS) and Symptom Checklist-90 were administered, as well as pandemic-related items. Overall, 1,775 patients (mostly married females with hypertension) were recruited through a community family physician group. Mean SSS scores were 36.1 ± 10.5/80, with 41.5% scoring in the elevated range (i.e., >36). In an adjusted model, being female, diagnosis of coronary artery disease and arrhythmia, perceived impact of pandemic on life, health condition, change to exercise routine, tolerance of control measures, as well as perception of future and control measures were significantly associated with greater distress. One-quarter perceived the pandemic had a permanent impact on their life, and 44.1% perceived at least a minor impact. One-third discontinued exercise due to the pandemic. While 47.6% stocked up on their medications before the lockdown, their supply was only enough for two weeks; 17.5% of participants discontinued use. Chief among their fears were inability to access healthcare (83.2%), and what they stated they most needed to manage their condition was medication access (65.6%). Since 2020 when we assessed a similar cohort, distress and perceived impact of the pandemic have worsened. Greater access to cardiac rehabilitation in China could address these issues.

## 1 Introduction

The coronavirus disease (COVID-19) pandemic, with its many unpredictable waves, has resulted in major negative impacts on economies, health systems, and citizens worldwide [1,2]. While the impact of COVID-19 on the health of all people has been of major concern, it has been a particular concern for those at higher risk of severe outcomes. This includes notably patients with chronic conditions, such as cardiovascular diseases (CVD). For instance, hospitalization rates and mortality rates are much higher in this population; in the United States alone, chronic diseases account for 75% of aggregate healthcare spending and are responsible for 7 out of 10 deaths [3]; in China, the deaths caused by chronic diseases account for 87% of total deaths [4].

Measures to control infection spread, such as physical distancing often necessitating closure of essential businesses [5], as well as diversion of healthcare resources for COVID-19 [6], have also had a negative impact. In the general population, this has resulted in reduced access to care [7] as well as increases in mental health conditions [8]. Despite their greater risk, there is only limited study of the impacts of the pandemic and associated control measures on the psychosocial well-being and self-management of patients with chronic conditions; we identified some abstracts only to date [9,10].

Government policy varies worldwide in terms of implementation of infection prevention and control measures. Based on the COVID-19 stringency index, such strategies are among the most stringent in China [11]. For example, in one of the world’s most populous cities of Shanghai, a strict “closed-loop” control system has been enforced. This involves home isolation except for medical reasons, with extra-household interpersonal contact and outdoor time forbidden, and transactions quickly moving online. This caused residents to become anxious and unsettled [12].

Given these unprecedented circumstances, assessment of the impacts of these strict control measures on chronic disease patients is needed, particularly in the context of some of the most stringent control measures globally, in one of the most densely populated cities in the world where risk of transmission is hence greater [13,14], and 2 years into the pandemic when these patients have thus been coping with isolation, economic impacts, as well as changed and inconvenient access to care and treatments for a prolonged period – specifically during the Omicron wave in Shanghai. Therefore, the objective of this study was to assess these impacts in terms of (1) psychosocial well-being, (2) health management, and (3) perceptions related to the pandemic and associated control measures. The associations of their health/well-being with the perceived impact of the pandemic on their health were also tested, as were the association of pandemic-related attitudes with psychological well-being.

## 2 Methods

### 2.1 Design and procedure

Study approval was secured from the Ethics Committee of Xinhua Hospital, affiliated with Shanghai Jiaotong University School of Medicine (XHEC-C-2022-042). The anonymous online survey in Simplified Chinese was created by the Cardiac Rehabilitation (CR) group, Health Risk Assessment and Control of the Chinese Preventive Medical Association. Data collection for this cross-sectional study was undertaken between March and June 2022. It was distributed via Wenjuanxing by family doctors who belonged to the Community Physician group of the Psychosomatic Medicine Special Committee of the Shanghai Association of Integrated Chinese and Western Medicine; this group cooperates closely with the Chinese Preventive Medical Association.

### 2.2 Setting and participants

During the Omicron wave lockdown in Shanghai, residents including patients with chronic conditions were to remain indoors and could not go outside to exercise or purchase groceries at the supermarket. They could receive care via telehealth or consultation in internet hospitals. Medication was available through online pharmacies with in-home delivery. This could be challenging for seniors who may have low digital literacy or who do not have access to a smartphone.

To receive in-person care, health codes had to be scanned. Daily nucleic acid tests were mandatory for in-patients and the one allowed family member who could accompany a patient.

Participant inclusion criteria were the following: outpatients with cardiovascular and cerebrovascular diseases, diabetes, kidney, or another chronic condition (e.g., cancer). Participant exclusion criteria were: (1) outpatients with severe cognitive impairments or any conditions that prevent them from being able to understand and agree to participate in the research; (2) outpatients unwilling to cooperate with the research protocol; and (3) outpatients with any missing data within questionnaire items.

### 2.3 Measures

All items were self-reported. Two relevant and validated psychosocial scales available for use in China were selected by the CR group, assessing depressive and anxiety symptoms, somatic symptoms, as well as sleep and cognitive issues. First, five items from the validated Symptom Checklist-90 were administered to assess depressive symptoms [15,16]. There is a 5-point Likert-type response option for each, with a maximal score of 25; higher scores indicate more severe symptoms.

The Chinese version of the Somatic Symptom Scale (SSS) was also administered [17]. It comprises 20 items, each assessed on a 4-point Likert-type scale. Items assess physical (e.g., half the items query each body system), depressive, and anxiety symptoms, and the final 2 items assess sleep and cognitive issues. Thus, the SSS assesses psychosocial well-being broadly. Total scores range from 20 to 80; scores ≥36 were considered elevated.

In addition, non-psychometrically validated items related to pandemic perceptions and health management were generated by the CR group, based on their knowledge and expertise. Response options were forced choice, with some where respondents could select all that apply. Participants were asked to respond these items based on their experience in the prior 2 weeks.

### 2.4 Statistical analysis

IBM SPSS statistics version 25.0 was used for all statistical analyses, with *p* < 0.05 considered statistically significant. Descriptive analysis was first performed. Perceptions of impact of the pandemic (at least minor vs none) on their chronic condition and psychosocial well-being were tested using chi-square and *t*-tests as applicable. The association of pandemic-related perceptions with SSS scores was assessed using the *t*-test and chi-square, as applicable. Finally, associations between participant sociodemographic and clinical characteristics with SSS scores were tested using logistic regression analysis as applicable. Then, an adjusted model was computed for the association of the SSS with those sociodemographic, clinical, and pandemic-related perceptions that were significant at *p* < 0.05.

## 3 Results

Of 1,775 responding patients, most were educated, working, married females living with family, and who had healthcare insurance (Table 1). As shown in Table 2, most participants had hypertension, over one-fifth had diabetes, and just under that coronary artery disease. Other conditions included cancer, as well as endocrine and immune diseases, and psychiatric conditions.

**Table 1:** Participant sociodemographic characteristics (*N* = 1775)

Characteristic	*n* (%)/mean ± SD
Sex (female)	1,061 (59.8)
Age	57.69 ± 13.80
Marital status	
Married	1,489 (83.9)
Widowed	111 (6.3)
Never married	107 (6.0)
Separated/divorced	68 (3.8)
Living situation	
With children and/or other extended family members	786 (44.3)
With partner only	768 (43.3)
Alone	221 (12.5)
Highest educational attainment	
Junior college or senior high school	818 (46.1)
Bachelor and above	723 (40.7)
Junior high school and below	234 (13.2)
Income status	
Has work income	1,501 (84.6)
No fixed income (e.g., rely on children, state subsidies)	138 (7.8)
Has additional source of income on top of salary (e.g., online)	136 (7.7)
Current or previous occupation	
City worker	557 (31.4)
Civil servants	440 (24.8)
Management (private or public)	289 (16.3)
Health care provider	181 (10.2)
Teacher	129 (7.3)
Government	59 (3.3)
Own business	44 (2.5)
Farmer	34 (1.9)
Research	34 (1.9)
Other (e.g., Arts)	8 (0.5)
Healthcare insurance	
Employee	960 (54.1)
Town residents	664 (37.4)
Off-site	77 (4.3)
Self-pay	74 (4.2)

SD, standard deviation.

**Table 2:** Participant physical and mental health and association with pandemic health impact (*n* = 1,775)

	*n* (%)/mean ± SD	Perceived impact of pandemic on health condition			
		No effect (*n* = 993)	Minor or greater (*n* = 782)	*X* ^2^/*t*	*p*
Chronic condition					
Coronary artery disease	333 (18.8)	132 (13.3)	201 (25.7)	44.209	<0.001
Heart failure	49 (2.8)	12 (1.2)	37 (4.7)	20.228	<0.001
Arrhythmia	189 (10.6)	75 (7.6)	114 (14.6)	22.693	<0.001
Hypertension	938 (52.8)	481 (48.4)	457 (58.4)	17.559	<0.001
Cerebrovascular disease	98 (5.5)	37 (3.7)	61 (7.8)	13.923	<0.001
Diabetes	405 (22.8)	210 (21.1)	195 (24.9)	3.565	0.059
Renal disease	62 (3.5)	23 (2.3)	39 (5.0)	9.259	0.002
Other	780 (43.9)	420 (43.7)	360 (44.2)	0.049	0.825
Years living with chronic condition					
<5 years	692 (39.0)	415 (41.8)	277 (35.4)	10.488	0.015
5–10 years	510 (28.7)	273 (27.5)	237 (30.3)		
10–20 years	344 (19.4)	173 (17.4)	171 (21.9)		
>20 years	229 (12.9)	132 (13.3)	97 (12.4)		
History psychiatric disorder					
Yes, being treated with meds	148 (8.3)	49 (4.9)	99 (12.7)	97.857	<0.001
Yes, but untreated	116 (6.5)	29 (2.9)	87 (11.1)		
Yes, but remitted	97 (5.5)	45 (4.5)	52 (6.6)		
No	1,414 (79.7)	870 (87.6)	544 (69.6)		
Major life event (*n*, % yes)	232 (13.1)	103 (10.4)	129 (16.5)	14.438	<0.001
Somatic self-rating scale (/80)	36.07 ± 10.50	31.38 ± 7.68	42.03 ± 10.58	−23.666	<0.001
Somatic symptoms (/36)	15.54 ± 4.65	13.52 ± 3.47	18.10 ± 4.69	−22.864	<0.001
Anxiety symptoms (/20)	8.84 ± 2.95	7.68 ± 2.18	10.30 ± 3.14	−19.925	<0.001
Depressive symptoms (/16)	7.59 ± 2.61	6.56 ± 2.12	8.90 ± 2.59	−20.437	<0.001
Sleep and cognitive symptoms (/8)	4.10 ± 1.36	3.62 ± 1.17	4.72 ± 1.35	−18.017	<0.001
SCL-90 depressive items (/25)	9.94 ± 4.29	8.35 ± 3.28	11.95 ± 4.57	−18.563	<0.001
Sadness (/5)	2.39 ± 1.02	2.07 ± 0.88	2.80 ± 1.03	−15.703	<0.001
Suicidal ideation (/5)	1.45 ± 0.80	1.21 ± 0.54	1.75 ± 0.96	−13.882	<0.001
Hopelessness (/5)	1.83 ± 1.02	1.50 ± 0.79	2.25 ± 1.12	−15.865	<0.001
Feel empty inside (/5)	2.04 ± 1.03	1.70 ± 0.85	2.47 ± 1.07	−16.400	<0.001
Anhedonia (/5)	2.23 ± 1.09	1.87 ± 0.93	2.69 ± 1.10	−16.622	<0.001

SD, standard deviation; SCL, symptom checklist 90.

Note: some items allowed single responses and others multiple.

The mean psychosocial well-being scores are shown in Table 2. Approximately 80% had no known psychiatric history. 41.5% of participants with elevated SSS scores indicating psychological distress (Table 3) was significantly more likely to be women, older, unmarried, living alone, with no fixed income, and had lower educational attainment than those with subclinical scores (all *p* ≤ 0.016). All chronic conditions except hypertension and diabetes were associated with significantly more distress on the SSS (all *p* ≤ 0.013), and they were living longer with their condition (*p* < 0.001). They were significantly more likely to have suffered a major life event (16.7% vs 10.5%; *p* < 0.001).

**Table 3:** Participant pandemic attitudes and impact on chronic disease management (*N* = 1,775)

	*n* (%)/mean ± SD	Association with somatic symptoms			
		Elevated (*n* = 736)	Subclinical (*n* = 1,039)	*X* ^2^/*t*	*p*
Source of Pandemic-related Information					
Traditional and new media (e.g., WeChat)	1,624 (91.5)	651 (88.5)	973 (93.6)	16.124	<0.001
Television and newspaper only (traditional media)	110 (6.2)	59 (8.0)	51 (4.9)		
No media, only family or neighbors	41 (2.3)	36 (3.5)	15 (1.4)		
Impact of pandemic on life					
None	210 (11.8)	42 (5.7)	168 (16.2)	200.903	<0.001
Only physical distancing	184 (10.4)	108 (14.7)	76 (7.3)		
Temporary	947 (53.4)	302 (41.0)	645 (62.1)		
Permanent changes	434 (24.5)	284 (38.6)	150 (14.4)		
View of pandemic status in future and control measures					
Situation is serious, and worry about future	652 (36.7)	378 (51.4)	274 (26.4)	144.777	<0.001
Think the strict measures will turn things around	403 (22.7)	144 (19.6)	259 (24.9)		
Always optimistic	397 (22.4)	85 (11.5)	312 (30.0)		
I am not worried about getting Omicron	323 (18.2)	129 (17.5)	194 (18.7)		
How long strict control measures can be tolerated					
I cannot even endure 1–2 weeks	166 (9.4)	111 (15.1)	55 (5.3)	65.327	<0.001
A month at maximum	847 (47.7)	367 (49.9)	480 (46.2)		
3 months maximum	258 (14.5)	79 (10.7)	179 (17.2)		
6 months maximum	23 (1.3)	9 (1.2)	14 (1.3)		
As long as I can get necessities, I can endure it for a long time	481 (27.1)	170 (23.1)	311 (29.9)		
Impact of pandemic on health condition^†^					
No effect	993(55.9)	214 (29.1)	779 (75.0)	407.066	<0.001
Minor	564 (31.8)	338 (45.9)	226 (21.8)		
Serious	218 (12.3)	184 (25.0)	34 (3.3)		
Adherence to control measures					
Stayed home	772 (43.5)	315 (42.8)	457 (44.0)	10.330	0.016
Only went out to get food and medicine	518 (29.2)	193 (26.2)	325 (31.3)		
Community sealed, so could not go out	460 (25.9)	215 (29.2)	245 (23.6)		
No change	25 (1.4)	13 (1.8)	12 (1.2)		
Exercise maintenance when strict control measures^†^					
Changed to exercise in home	761 (42.9)	240 (32.6)	521 (50.1)	58.656	<0.001
Stopped exercise because of pandemic	595 (33.5)	306 (41.6)	289 (27.8)		
Do not exercise as usual	366 (20.6)	169 (23.0)	197 (19.0)		
Exercise in residential area as before	53 (3.0)	21 (2.9)	32 (3.1)		
Access to medication when strict control measures					
I was sure to stock up before	845 (47.6)	306 (41.6)	539 (51.9)	50.597	<0.001
Someone else got my medication	360 (20.3)	165 (22.4)	195 (18.8)		
Discontinued use	310 (17.5)	170 (23.1)	140 (13.5)		
Internet dispensing	228 (12.8)	74 (10.1)	154 (14.8)		
Borrowed medicine from another	32 (1.8)	21 (2.9)	11 (1.1)		
Available supply of chronic disease medication					
I discontinued	236 (13.3)	116 (15.8)	120 (11.5)	66.290	<0.001
Not enough even for a week	309 (17.4)	168 (22.8)	141 (13.6)		
Enough for 1–2 weeks	469 (26.4)	213 (28.9)	256 (24.6)		
Enough for 2–4 weeks	393 (22.1)	138 (18.8)	255 (24.5)		
More than a month on hand	368 (20.7)	101 (13.7)	267 (25.7)		
Greatest worries about chronic condition related to Omicron					
Being unable to access care due to control measures	1,477 (83.2)	640 (87.0)	837 (80.6)	12.625	<0.001
Getting infected and deteriorating	747 (42.1)	372 (50.5)	375 (36.1)	36.913	<0.001
The economic impact of the pandemic affects health resourcing	438 (24.7)	190 (25.8)	248 (23.9)	0.878	0.349
Other	112 (6.3)	40 (5.4)	72 (6.9)	1.629	0.202
What needed during waves to manage chronic condition					
Easier access to medications	1,165 (65.6)	478 (64.9)	687 (66.1)	0.264	0.607
Ensuring access to care regardless of control measures	1,147 (64.6)	512 (69.6)	635 (61.1)	13.451	<0.001
Guarantee of emergency hospital and outpatient care access	992 (55.9)	450 (61.1)	542 (52.2)	14.079	<0.001
Facilitating access to care even if I cannot use or do not have a mobile phone	760 (42.8)	335 (45.5)	425 (40.9)	3.742	0.053
Greater availability of telehealth (year)	659 (37.1)	265 (36.0)	394 (37.9)	0.677	0.411

Note: some items allowed single responses and others multiple.

SD, standard deviation.

^†^Variables used for association tests in previous table.

### 3.1 Impact of pandemic

As shown in Table 3, most participants perceived the pandemic had a temporary impact on their lives, but almost one-quarter perceived changes would be permanent. In terms of the future, over one-third were very worried about it, and one-quarter each was optimistic or perceived the control measures would be successful in mitigating COVID-19 impact. Most commonly, participants reported that they could withstand the lockdown for a month.

Half perceived the pandemic had at least a minor impact on their health or more (Table 3). As shown in Table 2, those perceiving health impact were significantly more likely to be older, unmarried, and with lower educational attainment and income. Participants with cardiovascular, renal, and psychiatric diseases, who had their diseases for more years, reported major life events, and higher somatic and depressive symptoms (including all subscales) were also significantly more likely to perceive the impact of the pandemic on their health than their counterparts (Table 3).

Forty percent exercised at their home, but over one-third stopped exercising and one-fifth changed their exercise (Table 3). Participants who had their condition for fewer years (*p* = 0.009) and who had lower somatic (*p* < 0.001) were more likely to change their exercise due to the pandemic and associated control measures (no association with chronic condition).

With regard to medication, while almost half tried to stock up before the lockdown, almost one-fifth of participants discontinued use (Table 3). Only just over 10% of this older cohort used an internet-based pharmacy. Of those who still had medication, just under half had enough supply to last at least 2 weeks.

Participant’s greatest worries related to Omicron and their chronic condition are also shown in Table 3. Chief among them were fears around inability to access healthcare and medicines, as well as contracting COVID-19 and their health deteriorating. To best manage their chronic condition during the wave, participants most wanted guaranteed access to care and medication, particularly even if they do not have – or are not able to use – a mobile phone.

Pandemic perceptions were highly associated with SSS (Table 3). Participants who were significantly less likely to use new media to get pandemic-related information and more often relied on family or neighbours for information only and who were more likely to view pandemic impacts on their lives as permanent were less optimistic about the future, reported being able to tolerate control measures for a significantly shorter amount of time, perceived a significantly greater impact of the pandemic on their health, were less likely to go out even to get food or medicine, were more likely to stop exercise, and were more likely to discontinue medicine use or had less supply on hand, and had elevated SSS scores, indicating not only somatic but also anxious, depressive, and sleep-related symptoms. These participants with elevated distress were also more concerned about all but two of the worries related to the pandemic and were more likely to want assurances around access to in- and outpatient care regardless of COVID-19 wave status and associated control measures. An adjusted model is shown in Table 4.

**Table 4:** Model assessing correlates of SSS

Independent variable	OR	95% CI	*p*
Age	0.997	0.985–1.008	0.572
Sex			
Male	1		
Female	1.577	1.220–2.039	0.001
Marital status			
Married	1		
Widowed	0.898	0.502–1.606	0.716
Never married	0.808	0.446–1.463	0.481
Divorced	1.196	0.633–2.259	0.581
Living arrangement			
Alone	1		
With partner only	0.678	0.424–1.086	0.106
With children and/or other extended family members	0.677	0.438–1.045	0.078
Educational attainment			
Junior high school and below	1		
Junior college or senior high school	1.021	0.693–1.503	0.918
Bachelor and above	0.842	0.552–1.284	0.425
Income status			
No fixed income (e.g., rely on children, state subsidies)	1		
Has work income	0.696	0.433–1.118	0.134
Has additional source of income on top of salary (e.g., online)	0.630	0.330–1.203	0.161
Diagnosis			
Coronary artery disease	2.006	1.439–2.797	<0.001
Arrhythmia	2.588	1.731–3.870	<0.001
Pandemic media source type			
Traditional and new media (e.g., WeChat)	1		
Television and newspaper only (traditional media)	0.932	0.558–1.555	0.787
No media, only family or neighbors	1.139	0.432–3.002	0.793
Perceived impact of pandemic on life			
None	1		
Only physical distancing	2.566	1.491–4.418	0.001
Temporary	1.433	0.929–2.209	0.103
Permanent changes	3.298	2.051–5.305	<0.001
Perception of future and control measures			
Situation is serious, and worry about future	1		
Think the strict measures will turn things around	0.632	0.456–0.876	0.006
Always optimistic	0.418	0.291–0.600	<0.001
I am not worried about getting Omicron	0.636	0.453–0.894	0.009
Duration can tolerate control measures			
I cannot even endure 1−2 weeks	1		
A month at maximum	0.534	0.341–0.835	0.006
3 months maximum	0.415	0.245–0.706	0.001
6 months maximum	0.623	0.199–1.951	0.416
As long as I can get necessities, I can endure it for a long time	0.496	0.305–0.806	0.005
Adherence to control measures			
No change	1		
Only went out to get food and medicine	0.854	0.301–2.423	0.767
Stayed home	0.972	0.343–2.756	0.957
Community sealed, so could not go out	1.017	0.355–2.913	0.975
Perceived impact of pandemic on health condition			
No effect	1		
Minor	3.900	3.024–5.030	<0.001
Serious	12.905	8.340–19.968	<0.001
Change to exercise routine due to pandemic			
Do not exercise as usual	1		
Stopped exercise because of pandemic	0.850	0.609–1.187	0.340
Changed to exercise in home	0.596	0.432–0.822	0.002
Exercise in residential area as before	0.938	0.445–1.981	0.868

SSS: somatic self-rating scale, including physical disorders, depressive, anxious, sleep and cognitive symptoms as well.

## 4 Discussion

This is the first or one of the first full studies to examine the impact of the COVID-19 pandemic and its associated control measures in a large sample of older adults with chronic conditions [18]. It was undertaken during the Omicron wave in Shanghai, where the population is dense, control measures were strict, and the pandemic had been ongoing for 2 years. Despite that few were living alone that could mitigate isolation during the lockdown, consistent with findings in the general population [19,20], overall results show elevated distress but also negative impacts on chronic disease management. We must ensure that those with chronic conditions have the resources they need to cope and manage their condition, such as is offered in cardiac rehabilitation.

The burden of distress was quite high, even when considering that rates of psychological distress are higher in those with chronic disease [21,22] and rates in the Chinese population. Generally, rates of distress decline with age, but the opposite finding was found herein, likely due to the increased risk of severe COVID-19 with age [23,24,25]. Many patients were very concerned about the impact of the pandemic on their health and their access to care if needed. Research has shown that much preventive and non-preventive care was avoided due to the fear of exposure to SARS-CoV-2, and thus periodic health examinations regarding chronic disease risk factors were likely also missed, likely leaving weight [26], blood pressure, lipids, and blood glucose less-optimally managed.

In terms of health management, despite the proven benefits of secondary prevention behaviours, such as exercise and medication adherence [27], many chronic disease patients discontinued them. Despite the availability of online pharmacies and patient’s low supply of medication, these were not widely used, likely due to low digital literacy or technology access in the elderly [28,29]. One-third stopped exercising, and another quarter changed their exercise rather than moving their exercise routine home. How outpatients were able to maintain a healthy diet with dwindling food supplies would be another important area for study [30], as would be impacts of pandemic-related lockdowns on tobacco access and use in chronic disease patients. Moreover, given the importance of relationship quality to health [31], the impact of prolonged lockdown on relationships in chronic disease patients warrants study.

In our earlier examination of the impact of the pandemic and associated control measures on older adults (many of whom similarly had hypertension, CVD, or diabetes) in the spring of 2020 [32], mean scores on the SSS were somewhat lower at 29/80 vs 36 herein (all mean subscale scores were somewhat lower as well). In terms of pandemic impact on life, only 6% perceived this would be permanent in 2020 vs 25% in 2022. Also in 2020, most participants (47%) reported they could endure strict prevention and control measures for a long time (well over 6 months with another third saying they could endure 6 months); in 2022, however, almost 10% said they could not even endure strict prevention and control measures for a week or two and most (48%) reported that they could endure them for a maximum of a month. Also in 2020, 94% reported no effect of the pandemic on their health condition; yet, this was only 56% in 2022. Overall, for the common measures across the two surveys, perceptions worsened with time.

Study implications relate to ensuring that patients have what they need to manage their condition. What they most wanted was easier access to healthcare and medications, regardless of control measures. This is despite quite fast and broad availability of telehealth in China and online pharmacies [33]. This could likely be due to lower digital literacy or device availability in older adults, or preferences for in-person services [34,35]. Efforts to close the age-related “digital divide” must continue. Greater access to virtual cardiac rehabilitation in China could address these issues as well [6,36].

Caution is warranted when interpreting these results. First, this is a cross-sectional study, so the design precludes causal determinations. Second, the results may not be generalizable beyond China with its political and cultural context, and the city of Shanghai more specifically where the stringency of control measures was high. Nevertheless, the population of Shanghai is very large, and findings can inform other governments when considering their control measures. Moreover, similarity of the sample to the larger population is not known given the recruitment strategy, designed to quickly secure data during the lockdown, but where full reach and number of non-respondents were not collated. Third, all data were self-report, and hence, there may have been socially desirable responding or other measurement error. Relatedly, psychiatric conditions and health status were not verified through structured clinical interview or medical records. Fourth, due to the exploratory nature of the study, multiple comparisons were performed, increasing the chance of type 1 error. Finally, whether patients had COVID-19 prior to or during the period of study was not considered; more research is needed.

**Figure 1:**
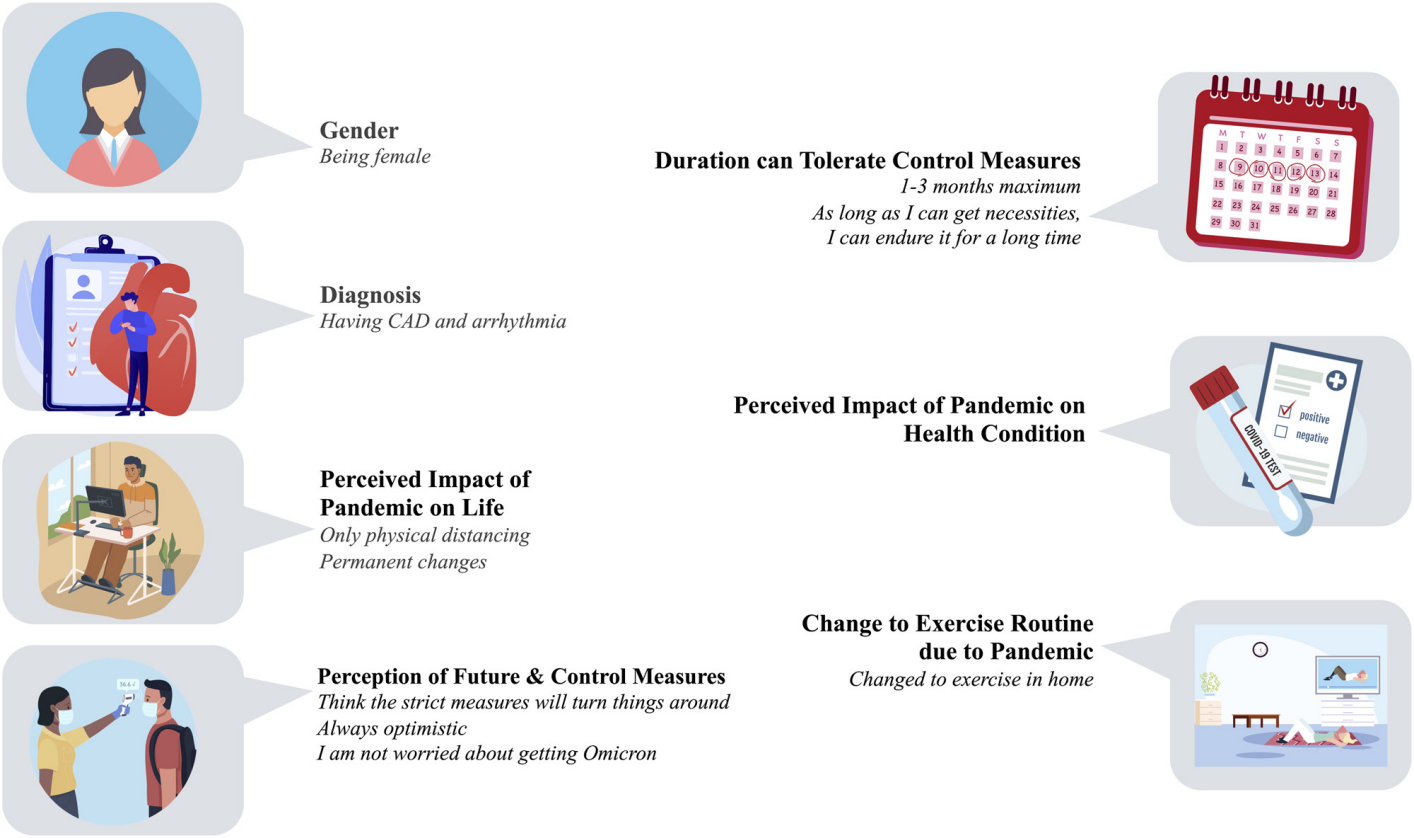
Correlates of psychosocial distress in chronic disease patients during stringent COVID-19 prevention and control measures. CAD: coronary artery disease.

In conclusion, 2 years into the pandemic in the midst of arguably the most stringent prevention and control measures globally, over 40% of chronic disease patients are experiencing elevated psychosocial distress, and this was associated with female, diagnosis of coronary artery disease and arrhythmia, perceived impact of pandemic on life, and impact of pandemic on health condition (Figure 1). Many patients stopped exercise and discontinued medication, and their greatest fears concerned access to healthcare. They were less able to tolerate prevention and control measures and had more negative perceptions related to the pandemic than at its beginning. Greater access to cardiac rehabilitation in China, including delivery supported by technology where patients are able, could address these issues.
